# Severe Doxycycline-Induced Oesophagitis With Granulomatous Inflammation Managed in a Rural Australian Hospital: A Case Report

**DOI:** 10.7759/cureus.119047

**Published:** 2026-11-03

**Authors:** Malcolm Han Tong Zou, Xinlin Chin, Jessica Y Ng

**Affiliations:** 1 General Surgery, Queensland Health, Queensland, AUS

**Keywords:** endoscopy, granulomatous reaction, oral doxycycline, pill-induced oesophageal injury, pill oesophagitis

## Abstract

Pill-induced oesophageal injury or pill oesophagitis typically presents with acute retrosternal pain, odynophagia, and dysphagia. While often self-limiting, cases may mimic acute coronary syndrome, food bolus, oesophageal perforation, and mediastinitis. Doxycycline is among the most commonly implicated medications. We report the case of a 32-year-old woman with severe mid-oesophageal ulceration secondary to doxycycline commenced for rosacea, managed at a rural Australian hospital. The histopathology finding of granulomatous inflammation was deemed reactive and secondary to severe ulceration, in the absence of infection or sarcoidosis. The patient was treated supportively with complete clinical and endoscopic resolution. Clinicians should consider pill oesophagitis in patients with acute retrosternal pain and odynophagia after starting doxycycline, and conservative management can be successful.

## Introduction

Pill oesophagitis occurs with delayed passage of medicines and prolonged exposure to oesophageal linings [1]. Over 100 medications have been implicated in inducing direct mucosal injury, including tetracyclines, bisphosphonates, potassium chloride, ferrous sulphate, and non-steroidal anti-inflammatory drugs [1,2]. Doxycycline is a second-generation tetracycline typically prescribed for acne, rosacea, and sexually transmitted infections [3-5]. Pill oesophagitis is characterised by abrupt retrosternal pain and odynophagia with or without dysphagia shortly after ingestion of medication [1]. Injury commonly affects the mid-oesophagus, where extrinsic compression from the aortic arch or left main bronchus may predispose to tablet retention [6-8]. Endoscopic findings typically include focal erythema, erosions, “kissing” ulcers, bleeding ulcers, or, rarely, strictures [6].

This case report aims to highlight the potential for doxycycline-induced oesophagitis to cause severe circumferential oesophageal injury requiring prolonged hospitalisation, multidisciplinary management, and investigation to exclude life-threatening complications. It also seeks to emphasise the importance of early recognition, timely endoscopic assessment, and effective conservative management in a regional Australian hospital, alongside preventative prescribing practices.

## Case presentation

A 32-year-old Australian-born woman presented to a rural emergency department with four days of retrosternal pain and foreign-body sensation. She reported pain at rest and severe odynophagia limiting oral intake. She had commenced oral doxycycline, 100 mg slow-release capsule daily, for rosacea seven days earlier. Her relevant history included endometriosis, irritable bowel syndrome, and a previous laparoscopic cholecystectomy. She underwent an unremarkable gastroscopy and colonoscopy six years ago. She took no other medications, was a non-smoker, and rarely consumed alcohol. On examination, there was mild epigastric tenderness, and she remained haemodynamically stable.

Initial blood tests demonstrated mild leukocytosis (11.4 × 10^9^/L) with neutrophilia (8.39 × 10^9^/L); serum lipase, liver function tests, and serial troponins were unremarkable (Table 1). Chest radiograph and serial electrocardiography traces were normal.

**Table 1:** Initial serum investigation results on presentation.

Serum investigation	Result	Reference range
White cell count (×10^9^/L)	11.4	4.0–11.0
Neutrophils (×10^9^/L)	8.39	2.0–8.0
Total bilirubin (μmol/L)	16	<20
Alkaline phosphatase (U/L)	94	30–110
Gamma-glutamyl transferase (U/L)	12	<38
Alanine transaminase (U/L)	12	<34
Aspartate transaminase (U/L)	18	<31
Lipase (U/L)	26	<60
Troponin I (ng/L)	3	<10
Troponin I repeated at 3 hours (ng/L)	2	<10

Doxycycline was immediately ceased. She commenced a clear fluid diet, intravenous pantoprazole 40 mg twice daily, antiemetics, and multimodal analgesia. Contrast-enhanced CT of the chest and abdomen with oral contrast showed mild wall thickening in the middle third of the oesophagus without extraluminal leak or mediastinal free air (Figures 1A, 1B).

**Figure 1:**
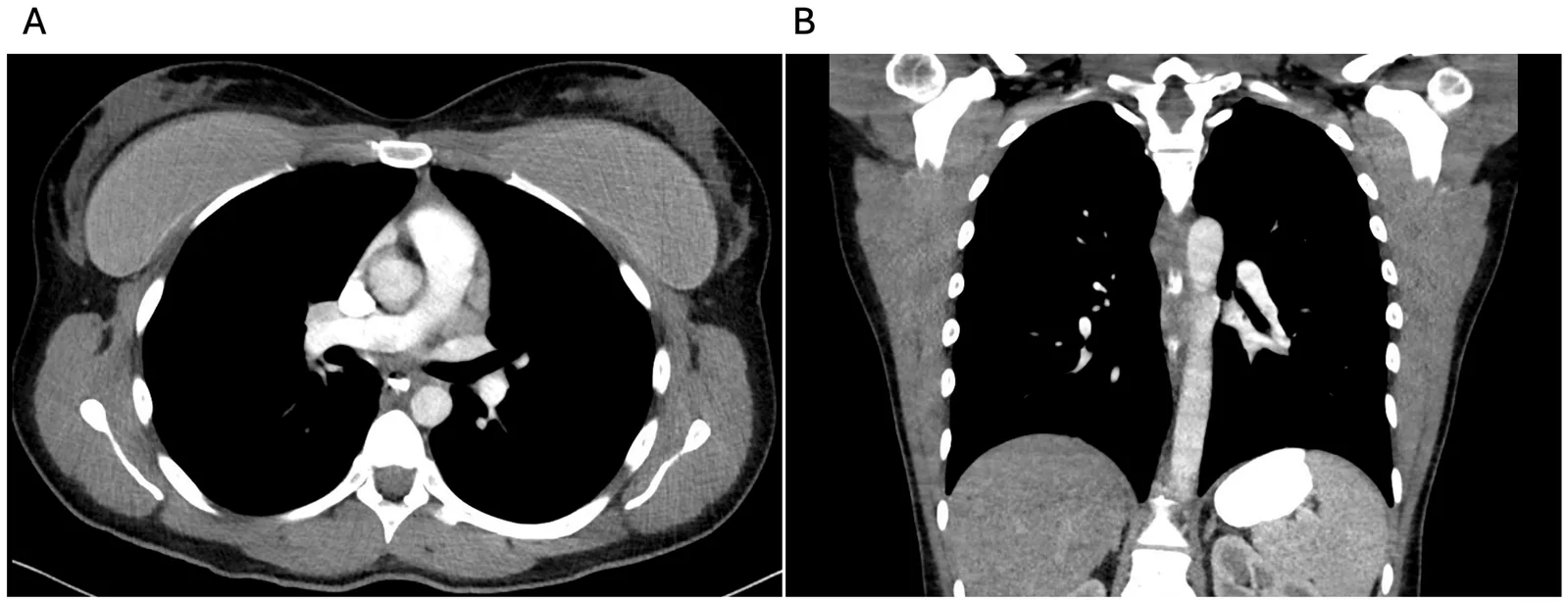
Initial CT of the chest and abdomen demonstrating mid-oesophageal thickening on axial (A) and corresponding coronal (B) slices.

Within 24 hours of presentation, she underwent an upper gastrointestinal endoscopy which demonstrated localised circumferential erosions in the middle third of the oesophagus (Figures 2A, 2B). The gastro-oesophageal junction was anatomically preserved, with a regular Z-line identified 34 cm from the incisors. Bilious fluid was observed within the stomach; however, the gastric cardia and fundus appeared normal on retroflexion, and the examined duodenum was unremarkable. Cold-forceps biopsies were obtained directly from the oesophageal lesion for histopathological and microbiological examination. The procedure was completed without immediate complications. Histopathology from the mid-oesophageal biopsy demonstrated ulcerative oesophagitis with granulomatous inflammation. There was no dysplasia, malignancy, viral inclusions, or foreign material. Immunostains for cytomegalovirus and herpes simplex virus were negative. She was commenced on an oral sucralfate suspension 1 g four times daily for one week.

**Figure 2:**
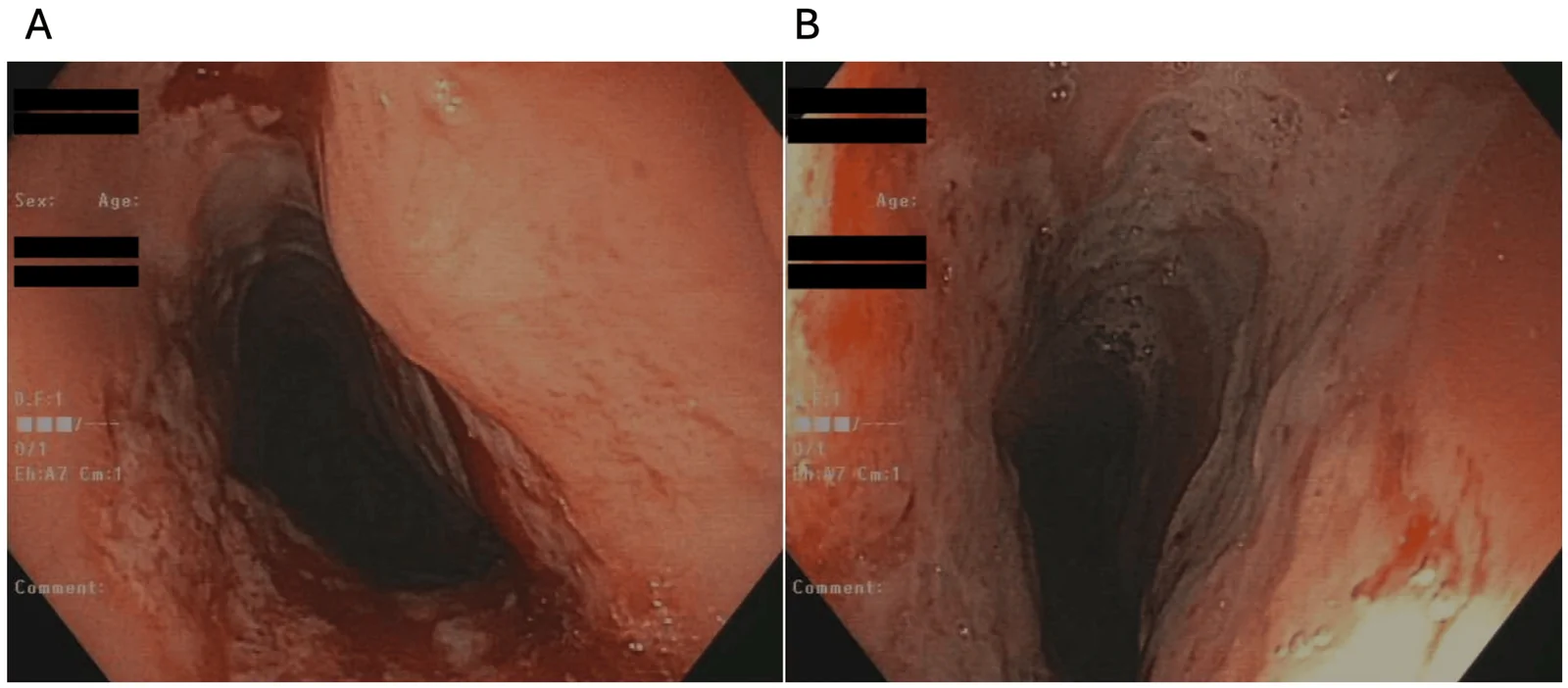
Large circumferential erosions within the middle third of the oesophagus on initial endoscopic assessment with images immediately proximal (A) and distal (B) to the impression from the left main bronchus.

With regular dietitian input, her diet was advanced to free fluids on day five, then soft diet on day seven of admission. By day eight, she was pain-free and discharged with oral pantoprazole 40 mg twice daily and instructed to remain upright during oral intake. She remained clinically well in the community, and repeat upper gastrointestinal endoscopy two months after the index admission demonstrated a normal oesophagus, stomach, and duodenum (Figures 3A, 3B).

**Figure 3:**
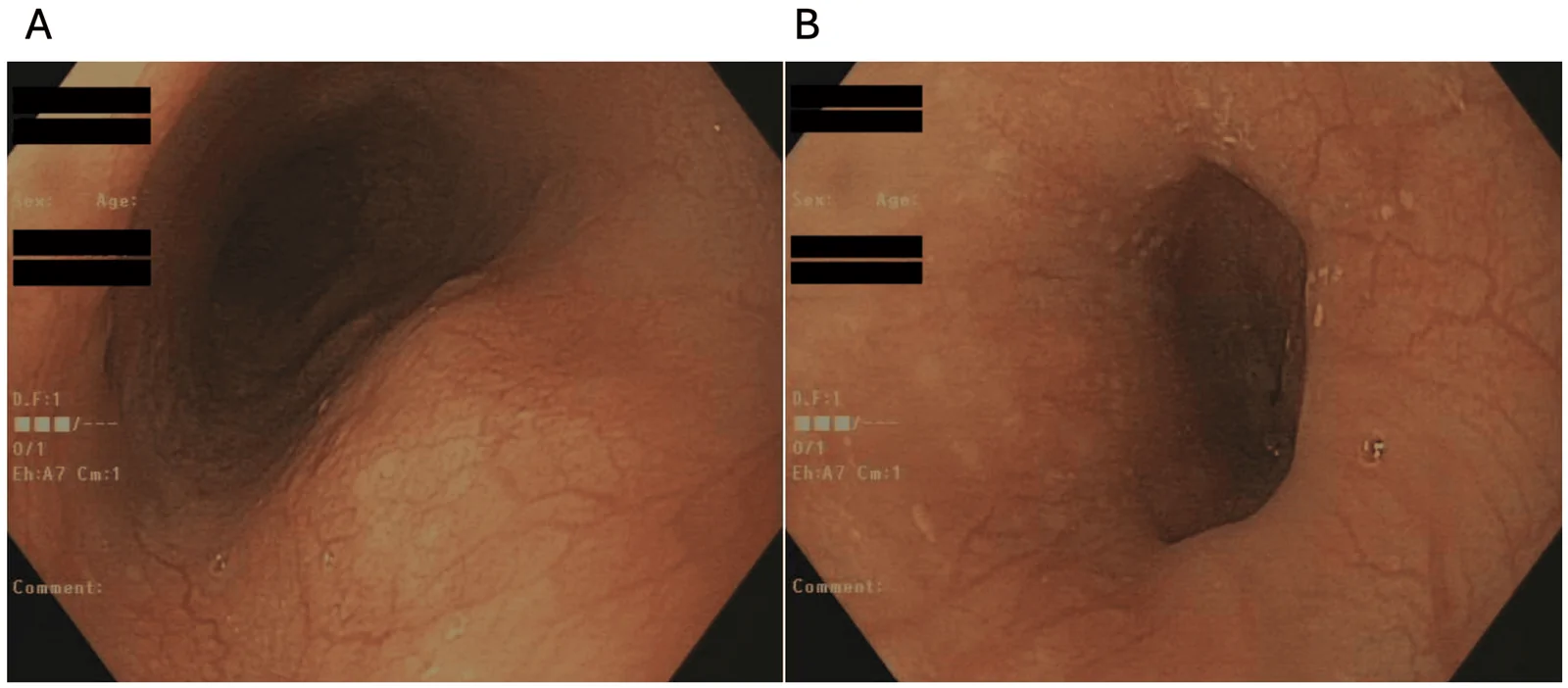
Complete resolution at the two-month follow-up endoscopy with images immediately proximal (A) and distal (B) to the impression from the left main bronchus.

## Discussion

Large clinical series have shown that pill oesophagitis commonly presents with chest pain, odynophagia, dysphagia, vomiting, haematemesis, and melaena [6,9]. In Kim et al.’s 78-patient series, 78.2% of lesions were located in the middle third of the oesophagus [9]. Endoscopic findings in that cohort were usually ulceration or erosion, consistent with the present case [9]. Doxycycline is repeatedly identified as a leading cause of pill oesophagitis. Al-Mofarreh and Al Mofleh reported 36 cases of oesophageal ulceration complicating doxycycline therapy in a predominantly young population [10]. Kadayifci et al. described five cases of doxycycline-induced pill oesophagitis, all in young or middle-aged women, with mid-oesophageal injury and retrosternal pain or dysphagia [11]. More recent series in patients receiving doxycycline for acne have reinforced the same pattern of abrupt symptoms, mid-oesophageal ulceration, and recovery after cessation of the drug [12].

In this case, the patient had recently commenced doxycycline capsules containing modified-release pellets of doxycycline hyclate, coated to slow but not prevent release in acid media [13]. These gelatin-coated capsules are likely more adherent to the oesophagus than similarly sized tablets, causing premature release of their contents [7]. This proposed mechanism of prolonged mucosal contact by the offending medication is exacerbated when ingested with insufficient water or immediately before lying down [1,6]. Doxycycline has acidic properties when dissolved locally, which may explain its capacity to produce sharply demarcated erosive or ulcerative injury [6]. The mid-oesophagus is vulnerable because of anatomical narrowing and extrinsic compression by the aortic arch and left main bronchus. Accounting for this, preventative education should include instructions to swallow pills with adequate water, remain upright after ingestion, and avoid lying down for at least 30 minutes.

Immediate management involves cessation of offending medication. Acid suppression is commonly used to reduce secondary reflux-mediated injury and assist symptom control. Sucralfate is intended to provide a protective mucosal barrier. Intravenous fluids, antiemetics, and multimodal analgesia are useful adjuncts if pain precludes adequate oral intake.

Severe persistent retrosternal pain can raise concern for perforation or mediastinitis. In this case, endoscopy confirmed partial-thickness mucosal injury, while CT with oral contrast was reassuringly negative for leak, free air, and mediastinal collection. This permitted ongoing conservative management in a rural hospital.

Histopathology in pill-induced oesophagitis is often non-specific but helps exclude malignancy, infection, eosinophilic oesophagitis, and other inflammatory mimics [8,14,15]. Kim et al.’s histomorphological study described features that may favour pill-induced injury over reflux oesophagitis, including diffuse dilated intercellular spaces, intraepithelial pustules, eosinophilic microabscesses, and inflammatory infiltrates [15]. There are broad-ranging differential diagnoses for granulomatous oesophagitis, including infection (tuberculosis, fungal, viral), sarcoidosis, foreign-body reaction, eosinophilic oesophagitis, and malignancy. In our case, the apparent granulomatous inflammation was unusual but not accompanied by acid-fast bacilli, fungal elements, viral inclusions, dysplasia, or foreign material, supporting a reactive inflammatory process secondary to severe ulceration.

General surgeons frequently assess patients with acute dysphagia and chest or epigastric pain in rural settings where formal gastroenterology services are not immediately available. Recognition of medication-induced oesophageal injury, early endoscopic assessment, and multidisciplinary management are key to achieving favourable outcomes.

This report is limited by its single-case design, which restricts generalisability, and the absence of formal causality assessment, drug rechallenge, or analysis of doxycycline formulation and drug concentration. Diagnosis was supported by temporal association, characteristic endoscopic findings, and clinical resolution following drug cessation. Furthermore, management within a regional Australian hospital may have been influenced by resource limitations and access to subspecialty services, potentially affecting the applicability of these findings to other healthcare settings.

## Conclusions

Doxycycline-induced oesophagitis should be considered in the differential diagnosis of patients presenting with acute retrosternal pain and odynophagia following recent initiation of doxycycline therapy. Severe cases may clinically mimic oesophageal perforation or food bolus impaction, potentially leading to diagnostic uncertainty. Upper gastrointestinal endoscopy plays a key role in establishing the diagnosis, evaluating the extent of mucosal injury, and excluding alternative oesophageal pathology. Early recognition and prompt discontinuation of the offending medication are essential for favourable clinical outcomes. Conservative management, including acid suppression, mucosal protection with sucralfate, and appropriate nutritional support, is generally effective. Furthermore, patient education regarding proper medication administration, particularly adequate water intake and remaining upright after ingestion, is crucial to preventing recurrence. This case highlights the importance of maintaining a high index of suspicion for medication-induced oesophageal injury to facilitate timely diagnosis, avoid unnecessary interventions, and optimise patient outcomes.
